# Glucocorticoid receptor gene polymorphisms associated with progression of lung disease in young patients with cystic fibrosis

**DOI:** 10.1186/1465-9921-8-88

**Published:** 2007-11-29

**Authors:** Harriet Corvol, Nadia Nathan, Celine Charlier, Katarina Chadelat, Philippe Le Rouzic, Olivier Tabary, Brigitte Fauroux, Alexandra Henrion-Caude, Josue Feingold, Pierre-Yves Boelle, Annick Clement

**Affiliations:** 1 Université Pierre et Marie Curie-Paris6, Paris, 75571 France; 2 Inserm, UMR-S 707, Paris, 75000 France; 3 AP-HP, Hôpital Trousseau, Pediatric Pulmonary Department, Paris, 75571 France; 4 AP-HP, Hôpital Trousseau, Genetics Department, Paris, 75571 France; 5 AP-HP, Hôpital St. Antoine, Biostatistics Department, Paris, 75571 France

## Abstract

**Background:**

The variability in the inflammatory burden of the lung in cystic fibrosis (CF) patients together with the variable effect of glucocorticoid treatment led us to hypothesize that *glucocorticoid receptor *(*GR*) gene polymorphisms may affect glucocorticoid sensitivity in CF and, consequently, may contribute to variations in the inflammatory response.

**Methods:**

We evaluated the association between four *GR *gene polymorphisms, *TthIII*, *ER22/23EK*, *N363S *and *BclI*, and disease progression in a cohort of 255 young patients with CF. Genotypes were tested for association with changes in lung function tests, infection with *Pseudomonas aeruginosa *and nutritional status by multivariable analysis.

**Results:**

A significant non-corrected for multiple tests association was found between *BclI *genotypes and decline in lung function measured as the forced expiratory volume in one second (FEV_1_) and the forced vital capacity (FVC). Deterioration in FEV_1 _and FVC was more pronounced in patients with the *BclI *GG genotype compared to the group of patients with *BclI *CG and CC genotypes (p = 0.02 and p = 0.04 respectively for the entire cohort and p = 0.01 and p = 0.02 respectively for F508del homozygous patients).

**Conclusion:**

The *BclI *polymorphism may modulate the inflammatory burden in the CF lung and in this way influence progression of lung function.

## Background

Cystic fibrosis (CF) is an autosomal recessive disorder that is caused by mutations in the CF transmembrane conductance regulator (*CFTR*) gene [1]. This gene encodes a protein that functions as a chloride channel in epithelial membranes [2]. Morbidity and mortality from CF is predominantly due to progressive loss of lung function, which follows a chronic course of inflammation, bacterial infection, and airway obstruction [3].

For a long time it was thought that the ineffective clearance of bacteria from the CF airways was primary to pathogenesis, leading secondarily to lung inflammation. However, there is now growing evidence that excessive inflammation is present very early in the airways, possibly even before infection [4]. The inflammatory response of the lung is persistently neutrophilic, with up-regulation of neutrophil chemotactic mediators such as interleukin (IL)-8 [5]. Accumulation of activated neutrophils with release of toxic products contributes to infection and subsequent chronic colonization by microorganisms such as *Pseudomonas aeruginosa *(*P. aeruginosa*) [6]. Amplification of the vicious cycle of inflammation/infection leads to progressive lung destruction.

As inflammation is a central contributor to the pathogenesis of CF pulmonary disease, limiting the excessive production of inflammatory mediators represents a major therapeutic strategy to slow the decline in lung function and to improve survival [7]. In this context, glucocorticoids are an obvious choice due to their wide range of anti-inflammatory effects, particularly on neutrophils [8]. Although beneficial, the use of systemic glucocorticoids is limited by their unacceptable side effects [9,10]. Inhaled glucocorticoids offer the possibility of increased airway deposition together with fewer systemic effects, explaining the dramatic increase in their use in CF in recent years [11]. However, the effectiveness of regular use of inhaled glucocorticoids in the management of patients with CF remains uncertain based on the results of the various trials in which inhaled glucocorticoids were compared to either placebo or standard treatment [12,13]. Indeed, no convincing conclusions could be drawn, as the reported studies were heterogeneous with respect to inclusion criteria, age, severity of pulmonary involvement, as well as type and duration of treatments. Recently, Balfour-Lynn and co-workers performed a multicenter trial to test the hypothesis that withdrawing inhaled glucocorticoids would not be associated with an earlier onset of acute chest exacerbations [14]. Their results support the conclusion from the Cochrane review that there is evidence of neither benefit nor harm, and that, most likely, specific subgroups of patients with CF may benefit from inhaled glucocorticoids [11]. Indeed, evidence indicating that significant variation among individuals to therapeutic glucocorticoids, with regard to disease response and to susceptibility to glucocorticoid side effects, exists [15,16].

**Figure 1:**
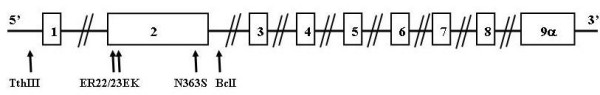
Positioning of the *glucocorticoid receptor *gene polymorphisms studied in the cystic fibrosis patients.

The clinical course in CF is highly variable, and compelling information on phenotypic variability and lack of genotype-phenotype correlation among patients with the same mutation in the *CFTR *gene has led to suggest that modifier genes affect the CF phenotype [17]. Recently, polymorphisms in candidate genes involved in the inflammatory cascade have been shown to modulate the expression of the clinical phenotype [18,19]. In several inflammatory diseases, variations in glucocorticoid sensitivity have been reported to be associated with single nucleotide polymorphisms (SNP) in the *glucocorticoid receptor *(*GR*) gene [16,20-22]. Among them, a *GR *polymorphism in exon 2 (*N363S*), which alters the N-terminal transactivation domain, was described to be associated with glucocorticoid hypersensitivity [20]. Another polymorphism was identified in exon 2. It comprises 2 point mutations in codons 22 and 23, and the relevant the *ER22/23EK *allele being linked to a decrease in the response to dexamethasone [21]. The *TthIII *polymorphism in the 5' untranslated region was found to be associated with basal cortisol secretion and the *BclI *polymorphism, located in intron 2, with an increased sensitivity to corticosteroids [22,23] (Figure 1).

The variability in the inflammatory burden of the lung in CF patients together with the variable effect of glucocorticoid treatments led us to hypothesize that *GR *gene polymorphisms may affect glucocorticoid sensitivity in CF and, consequently, may contribute to variations in the inflammatory response. In the present study we therefore evaluated the association between *GR *gene polymorphisms and disease progression in a cohort of young patients with CF.

## Methods

### Patients

The study population consisted of 255 young CF patients who attended 6 French CF care centers with similar patient management (CF centers of Trousseau children hospital and Debre hospital in Paris and CF centers of Caen, Rennes, Rouen and Toulouse). The diagnosis of CF was confirmed on the basis of 2 positive sweat chloride tests (> 60 mmol/L) and identification of mutations in the *CFTR *gene. Over a one-year period (January to December 2005), all the patients with pancreatic insufficiency were proposed to participate in the study during their regular outpatient visits to the CF centers, and a written informed consent was obtained from each patient and/or his/her parents. The study was approved by the French ethics committee of the St. Louis Hospital (Paris). Clinical, biological and functional data were obtained retrospectively from hospital records, blinded for the results of *GR *genotypes. Recorded data included sex, *CFTR *mutations, pulmonary function tests, airway microbiology, nutritional status, impaired glucose tolerance and diabetes. Lung function was assessed by measurement of forced expiratory volume in one second (FEV_1_) and forced vital capacity (FVC) in children > 6 yrs during periods of clinical stability. For each parameter (both FEV_1 _and FVC) the best value of three measurements was recorded, according to the guidelines of the European Respiratory Society and American Thoracic Society [24]. FEV_1 _and FVC were expressed as percentages of predicted normal values. Respiratory microbial flora was determined by microscopy and culture of lower respiratory tract secretions or throat swaps. Chronic airway infection with *Pseudomonas aeruginosa *(*P. aeruginosa*), a major cause of morbidity and mortality in CF, was defined by the persistence of the pathogen in at least three airway samples for at least 6 months. Nutritional status was appreciated by the *z*-score for the body mass index (BMI). Impaired glucose tolerance and diabetes were assessed by an annual oral glucose tolerance test in children > 10 yrs during periods of clinical stability according to the World Health Organisation criteria [25].

### Genotyping

Genomic DNA was extracted from blood samples using the QIAmp DNA Blood Kit (Qiagen, Courtaboeuf, France). *TthIII*, *ER22/23EK*, *N363S *and *BclI *genotypes were obtained with the fluorogenic 5' nuclease TaqMan^® ^Probe-based chemistry. First, the Polymerase Chain Reaction (PCR) in 96-well format was performed with a GeneAmp 2700 PCR system (Applied Biosystems, Foster City, USA) using TaqMan^® ^probes and primers designed by Applied Biosystems.

For *TthIII *(rs10052957), the forward primer was 5'-GCAGAGGTGGAAATGAAGGTGAT-3', and the reverse primer was 5'-GGAGTGGGACATAAAGCTATGACAA-3'. The probe corresponding to the reference allele (labeled with fluorescent FAM) was ATTCAGACTCAGTCAAGG. The probe corresponding to the variant allele (labeled with fluorescent VIC) was TATTCAGACTCAATCAAGG.

For *ER22/23EK *(rs6189 and rs6190), the forward primer was 5'-TCCAAAGAATCATTAACTCCTGGTAGA-3', and the reverse primer was 5'-GCTCCTCCTCTTAGGGTTTTATAGAAG-3'. The probe corresponding to the reference allele (labeled with fluorescent VIC) was ATCTCCCCTCTCCTGAG. The probe corresponding to the variant allele (labeled with fluorescent FAM) was ATCTCCCTTTTCCTGAGCA.

For *N363S *(rs6195), the primer sequences were not supplied by Applied Biosystem. The probe corresponding to the reference allele (labeled with fluorescent FAM) was TCCAGATCCTTGGCACCTATTCCAATTTTCGGAACCAACGGGAATT. The probe corresponding to the variant allele (labeled with fluorescent VIC) was TCCACATCCTTGGCACCTATTCCAACTTTCGGAACCAACGGGAATT.

For *BclI *(rs not available), the forward primer was 5'-CAGGGTTCTTGCCATAAAGTAGACA-3', and the reverse primer was 5'-GCACCATGTTGACACCAATTCC-3'. The probe corresponding to the reference allele (labeled with fluorescent FAM) was CTCTTAAAGAGATTCATCAGC. The probe corresponding to the variant allele (labeled with fluorescent VIC) was CTCTTAAAGAGATTGATCAGC.

The PCR conditions and cycling followed the manufacturer's instructions. Allelic discrimination was performed by endpoint measurements on the 7500 Real Time PCR System (Applied Biosystems). Genotyping data were collected for statistical analysis.

### Statistical analysis

Statistical analyses were performed for the entire cohort and in the subgroup of patients F508del homozygous. Data were expressed as a percentage, mean (± SD) or median [interquartile range]. Conformance of the allele frequencies with the Hardy-Weinberg equilibrium was tested using a using Fisher's exact test. For SNP *ER22/23EK *and *N363S*, the least frequent homozygous type was grouped with the heterozygous type for the statistical analyses. SNP pairwise linkage disequilibrium was evaluated by Lewontin's D'. Haplotypes were reconstructed with the EM algorithm and only the most probable resolution was retained for each child [26,27]. Haplotypes with frequencies <1% were grouped with the most frequent haplotype.

The association between the *GR *genotype and time to first *P. aeruginosa *infection was evaluated with proportional hazards regression models. Univariable and multivariable analyses were performed. Associations with the following patient characteristics were tested with the log likelihood ratio test: gender, circumstances of CF diagnosis (neonatal screening, meconium ileus, later diagnosis on clinical symptoms), birth date (cohort effect), CF centre, *CFTR *genotype, and pancreatic status.

Longitudinal linear mixed effect models were applied to FEV_1_, FVC and BMI z-score data to determine the patterns of change in pulmonary function and nutritional status with age and genotype. The mixed effect model takes into account the correlation within observations measured for the same subject. On the regression line linking FEV_1_, FVC or BMI to age, both the intercept (at 5 years) and slope could depend on the genotype, or on the count of haplotypes. Measurements between age 6 and 16 were used in the analyses. All models were adjusted at maximum likelihood, and the influence of genotype was tested by the Wald test. A single step permutation based correction for multiple SNP testing was made as described by Westfall and Young [28]. For each of 5000 surrogate datasets obtained by permuting individual genotypes, mixed model analysis was applied to the 4 SNP and the minimum P-value was recorded. The corrected P-value corresponds to the probability of finding a smaller P-value in the permutations.

Statistical significance was defined as *P *< 0.05. Statistical analyses were carried out with the R software (v2.4.0).

## Results

### Patient clinical characteristics

The clinical characteristics of the study population are listed in Table 1. The mean age at enrollment was 11.1 ± 5.1 years and the mean duration of follow-up was 11.2 ± 6.0 years. 136 out of the 255 patients were *F508del *homozygous and 91 *F508del *compound heterozygous. Among the other *CFTR *mutations, the most frequent were *G542X*, *G551D*, *I507del*, *N1303K *and *1717-1G>A*. 60% of the patients were chronically colonized with *P. aeruginosa*. As the average age of onset of diabetes in CF is 18–21 years, the number of patients with impaired and diabetic glucose tolerance in this paediatric cohort was relatively low; 21 with impaired glucose tolerance and 15 with diabetes.

**Table 1:** Clinical characteristics of cystic fibrosis patients

**Study population **(n)	255
Age at enrolment, yrs (mean ± SD)	11.1 ± 5.1
Age at diagnosis, yrs (mean ± SD)	1.4 ± 2.3
Sex, male/female	134/121
*CFTR *genotype	
*F508del *homozygous	136 (53%)
*F508del *compound heterozygous	91 (36%)
Other mutations	28 (11%)
*P. aeruginosa *infection at 9 yrs *	63 ± 3%
*P. aeruginosa *chronic colonization at 9 yrs*	60 ± 3%
Impaired glucose tolerance	21 (8%)
Diabetes	15 (6%)

Abbreviations: yrs: years; *P. aeruginosa*: *Pseudomonas aeruginosa*

* Cumulated incidence by Kaplan-Meier estimate

### Allele frequencies of *GR *polymorphisms

The genotype distributions in the studied patients are listed in Table 2. All the 255 patients have been genotyped for the 4 polymorphisms studied. The frequencies of the tested alleles and genotypes were similar to reported frequencies in control populations [20,22,29,30]. The population did not deviate significantly from the Hardy-Weinberg equilibrium indicating no bias in sampling in the examined population or problems with genotyping of the collected samples, and reflecting a random allele distribution.

**Table 2:** Genotype frequencies and p-value for Hardy-Weinberg equilibrium (HWE) in the cystic fibrosis patients. The genotype frequencies from previous studies in Caucasians are listed for comparison.

**Genotypes**	**Frequencies in the cystic fibrosis patients (n = 255)**	**Frequencies in previous studies in Caucasians**
***TthIII***		van Rossum *et al*. [30] (n = 209)
**TT**	0.09	0.16
**CT**	0.45	0.44
**CC**	0.46	0.40
**HWE**	0.66	
		
***ER22/23EK***		van Rossum *et al*. [21] (n = 202)
**AA**	0.004	0
**AG**	0.026	0.09
**GG**	0.97	0.91
**HWE**	0.30	
		
***N363S***		Huizenga *et al*. [20] (n = 216)
**GG**	0	0
**AG**	0.06	0.06
**AA**	0.94	0.94
**HWE**	0.61	
		
***BclI***		Rosmond et al. [45] (n = 284)
**GG**	0.10	0.14
**CG**	0.45	0.46
**CC**	0.45	0.40
**HWE**	0.79	

### Analysis of changes in respiratory parameters with *GR *genotypes

Changes in lung function (FEV_1 _and FVC) were examined for each genetic locus. The decline in FEV_1 _and FVC was analyzed by linear mixed model regression. This model predicts for the entire cohort a mean (± SD) decline per year in FEV_1 _of 2.3 (± 0.3)%, and in FVC of 1.7 (± 0.3)%. The decline in FEV_1 _and FVC with *GR *genotype as covariate was analyzed in the total population and in the subpopulation of F508del homozygous patients. The estimated values at 6-year and slopes in change per year are shown in Table 3 for the entire cohort and for the homogeneous subgroup of F508del homozygous patients. Deterioration in FEV_1 _in the entire cohort was more pronounced in patients with the *BclI *GG genotype (annual slope of decline, k: -3.4 ± 0.5) compared to the group of patients with *BclI *CG and CC genotypes (annual slopes of decline, k: -2.8 ± 0.7 and -2.3 ± 0.5 respectively, p = 0.02). This association was also significant in the homogeneous subgroup of F508del homozygous patients with a more pronounced rate of decline in FEV_1 _in patients with the *BclI *GG genotype compared to patients with *BclI *CG and CC genotypes (p = 0.01). Similarly, analysis of FVC data showed a more severe decline in patients carrying the *BclI *GG genotype compared to *BclI *CG and CC genotypes in the entire cohort and in the F508del homozygous patients (p = 0.04 and p = 0.02 respectively). After correction for multiple testing, these associations were borderline significant in the entire cohort and in the F508del homozygous patients (p = 0.07 and p = 0.06 respectively for FEV_1_).

**Table 3:** Analysis of *glucocorticoid receptor *genotypes and longitudinal trends for FEV_1 _and FVC

		**Total population**			***F508del/F508del *patients**		
	**Genotype**	Y_6 _(± SD)	k (± SD)	p	Y_6 _(± SD)	k (± SD)	p

**FEV_1_**							
***TthIII***	**CC**	95.5 (± 2.9)	-2.5 (± 0.5)	0.7	89.2 (± 4.1)	-2.2 (± 0.7)	0.4
	**CT**	96.2 (± 4.1)	-2.9 (± 0.7)		98.7 (± 5.4)	-3.8 (± 0.9)	
	**TT**	88.8 (± 6.7)	-1.6 (± 1.1)		94.4 (± 8.4)	-2.2 (± 1.3)	
							
***ER22/23EK***	**GG**	95.2 (± 11.2)	-2.6 (± 1.7)	0.4	94.6 (± 21.4)	-3.0 (± 2.9)	0.7
	**AA + AG**	92.6 (± 11.0)	-0.5 (± 1.7)		90.4 (± 21.2)	-0.9 (± 2.8)	
							
***N363S***	**AA**	95.5 (± 7.3)	-2.6 (± 1.4)	0.5	95.3 ± (9.9)	-3.0 (± 2.2)	0.5
	**GG + AG**	89.9 (± 7.0)	-2.9 (± 1.4)		85.3 ± (9.6)	-3.0 (± 2.2)	
							
***BclI***	**CC**	96.4 (± 2.8)	-2.3 (± 0.5)	0.02*	92.0 (± 3.7)	-2.3 (± 0.6)	0.01*
	**CG**	90.7 (± 3.9)	-2.8 (± 0.7)		89.3 (± 5.2)	-3.1 (± 0.8)	
	**GG**	106.8 (± 6.4)	-3.4 (± 1.2)		111.0 (± 7.2)	-4.3 (± 1.3)	

**FVC**							
***TthIII***	**CC**	97.6 (± 2.6)	-2.3 (± 0.4)	0.8	92.1 (± 3.7)	-1.8 (± 0.6)	0.9
	**CT**	93.8 (± 3.5)	-1.7 (± 0.6)		95.2 (± 5.0)	-2.2 (± 0.8)	
	**TT**	92.3 (± 5.9)	-1.6 (± 0.9)		92.8 (± 7.8)	-1.8 (± 1.3)	
							
***ER22/23EK***	**GG**	95.6 (± 9.7)	-2.1 (± 1.4)	0.1	93.8 (± 19.4)	-2.2 (± 2.7)	0.5
	**AA + AG**	86.4 (± 9.5)	+1.0 (± 1.4)		89.4 (± 19.3)	0.6 (± 2.6)	
							
***N363S***	**AA**	95.2 (± 6.3)	-1.9 (± 1.2)	0.9	94.1 (± 8.9)	-2.1 (± 2.0)	0.8
	**GG + AG**	94.9 (± 6.1)	-2.2 (± 1.2)		88.4 (± 8.6)	-1.7 (± 2.0)	
							
***BclI***	**CC**	96.0 (± 2.5)	-1.5 (± 0.4)	0.04*	91.7 (± 3.3)	-1.3 (± 0.4)	0.02*
	**CG**	92.0 (± 3.5)	-2.1 (± 0.6)		89.3 (± 4.7)	-2.1 (± 0.8)	
	**GG**	104.0 (± 5.7)	-2.8 (± 0.1)		107.8 (± 6.6)	-3.6 (± 1.2)	

Abbreviations: Y: years; FEV_1_: forced expiratory volume in 1 second; FVC: forced vital capacity. FEV_1 _and FVC are expressed as percentages of predicted values.

Statistical analysis: Mixed model regression for FEV_1_, FVC according to age. Results are expressed as estimated value at 6 years (Y_6 _± SD) and average decline per year (k ± SD). Mixed model equation is expressed as Y_age _= Y_6 _- *k *(age - 6). *p < 0.05.

For the *ThIII*, *ER22/23EK *and *N363S *variants, analysis of FEV_1 _or FVC data revealed no significant difference between the different genotypes.

In a Cox regression model, we did not find any significant association between the age of the first *P. aeruginosa *infection or acquisition of colonization and either *ThIII*, *ER22/23EK*, *N363S *or *BclI *variants (data not shown).

### Analysis of changes in respiratory parameters with *GR *haplotypes

Linkage disequilibrium between pairs of *GR *alleles (*TthIII*, *ER22/23EK*, *N363S *and *BclI*) was analyzed using D' coefficient (Lewontin's standardized disequilibrium coefficient). The D' values for the pairs were indicative of linkage disequilibrium (D' values between 0.17 to 0.99, and p values between 0.05 to <0.0001), leading to extend the study to haplotypes across the *GR *gene.

The reconstructed haplotype frequencies for *TthIII*, *ER22/23EK*, *N363S *and *BclI *were calculated. We found that the 4 single-nucleotide polymorphisms were segregated as 6 distinct haplotypes, with frequencies listed in Table 4. The most frequent haplotype was *TthIII/C *– *ER22/23EK/G *– *N363S/A *– *BclI/C*. Haplotype trend regression was used to associate *GR *haplotypes with changes in FEV_1 _and FVC but no significant association could be documented.

**Table 4:** Haplotype frequencies in the *glucocorticoid receptor *gene

***TthIII***	***ER2223EK***	***N363S***	***BclI***	**Frequency**
C	G	A	C	0.47
C	G	A	G	0.19
C	G	G	C	0.03
T	A	A	C	0.02
T	G	A	C	0.15
T	G	A	G	0.14

### Influence of the GR genotype on nutritional parameters

The decline in the BMI z-score, from 6 to 16 years was analyzed by mixed model regression. For *BclI *genotypes, the annual rates of decline in the BMI z-score were -0.04 ± 0.02 for *BclI *CC and CG and -0.05 ± 0.04 for *BclI *GG (p = 0.7).

No significant association was found between either *ThIII*, *ER22/23EK*, *N363S *or *BclI *variants and the nutritional parameters tested, which included decline in the BMI z-score, impaired glucose tolerance and diabetes.

## Discussion

A novel finding of the present study is that *BclI *polymorphism in the *GR *gene seems to be associated with lung disease progression in CF. Indeed, analysis of pulmonary function data showed a more pronounced rate in decline in patients carrying the *BclI *GG genotype. These observations are consistent with the effect of *GR *polymorphism on gene products and the role of *GR *in the control of the inflammatory response of the lung in CF.

Despite the monogenic nature of CF, the clinical course in this disease is highly variable in patients with identical *CFTR *genotypes [17]. Although environmental factors may contribute to this variation, several host genetic factors that code outside of *CFTR *locus have been reported to modulate the expression of several clinical phenotypes. Identification of modifier genes that may influence disease progression is becoming an important challenge in CF not only to progress in the understanding of CF pathophysiology but also to identify the patients who may benefit from new therapeutic strategies and to adapt the treatment according to the patient's genetic profile. Among the candidate genes of interest are the genes that can interfere with the inflammatory cascade and the response to anti-inflammatory agents [18,19].

Genes that influence the exogenous as well as endogenous glucocorticoid effects have been examined as potential modifiers in the present study. The key contributor to glucocorticoid action is GR, a member of the steroid-hormone-receptor family of proteins. Within the cell, the cortisol-GR complex can bind as a homodimer to the glucocorticoid-response elements and enhance or represses transcription of specific target genes [31]. The complex can also interact with other transcription factors such as nuclear factor-κB [32]. Other modes of action include glucocorticoid signaling through membrane associated-receptors. Therefore, it is currently believed that GR could inhibit inflammation through various genomic and non-genomic mechanisms. To date only one gene has been identified for GR, but several GR isoforms are generated by alternate splicing, alternative translation initiation, and each isoform is subject to a variety of post-translational modifications which play an important role in the subcellular distribution, protein turnover and transcriptional activities of GR [33]. In addition to the complexity of the multiple isoforms, *GR *mutations and polymorphisms may also affect protein expression, structure and function, and thus may have diverse clinical consequences.

Several SNP in the *GR *gene have been reported to be associated with variation in glucocorticoid sensitivity, mainly the *N363S*, *ER22/23EK*, *TthIII *and the *BclI *polymorphisms [20,22,29,30]. The *N363S *polymorphism is a non synonymous SNP and the *363S *allele has been associated with a higher BMI, enhanced cortisol suppression and an increased insulin response after dexamethasone administration [20]. The *ER22/23EK *polymorphism consists of two linked point mutations, the first is synonymous while the second is non synonymous. The *ER22/23EK *variant allele has been reported to be associated with a greater sensitivity to insulin, lower total and low-density lipoprotein cholesterol levels, and a beneficial body composition at a young adult age [21,29,34]. The *TthIII *polymorphism has been shown to be associated with changes in basal cortisol secretion in men [23]. The *BclI *polymorphism previously characterized as the large (4.5 kb) and small (2.3 kb) restriction fragments has recently been identified as a C to G mutation in intron 2, 646 bp downstream from exon 2 [22]. Several investigations have found association between the large allele or the GG genotype and parameters indicative of insulin resistance. As this *BclI *polymorphism is intronic, its effect on *GR *gene activity may be indirect. It is currently suggested that this polymorphism might affect the *GR *gene promoter by selectively acting either on repressor or enhancer sites.

The mechanisms by which the *BclI *polymorphisms could influence lung function and lung disease progression in CF remain to be elucidated. One explanation may be drawn from the results of several studies showing that this polymorphism influences glucocorticoid sensitivity [22,35-37]. In a number of chronic inflammatory disorders, evidence of decreased glucocorticoid sensitivity of blood cells has been reported [38-40]. The cause of this reduction remains at present unknown, but might, at least, in part, be genetically determined. As indicated above, the *BclI *polymorphism could affect differently the *GR *promoter leading to differences in receptor expression levels. By interacting with either repressor or enhancer sites within the promoter, glucocorticoid sensitivity would be increased or decreased. Differential usage of the promoter is likely to contribute to a variable response in a tissue-specific manner. This is supported by the data provided by Panarelli and coworkers in normal human subjects [37]. These authors showed that the skin sensitivity to budesonide was enhanced in subjects carrying the GG variants of the *BclI *polymorphisms compared with the CC subjects. In contrast, white blood cells of the GG subjects tended to be less sensitive to dexamethasone *in vitro*. Although these findings were not statistically significant, the authors suggested that this polymorphism might have tissue-specific effects and that the *GR *genotype could affect steroid sensitivity in a tissue-specific manner. As the inflammatory process in CF is dominated by a neutrophil influx in the airways, our present findings reporting that CF patients carrying the *BclI *GG genotype showed a more severe decline in lung function may be consistent with the findings of Panarelli and coworkers with a decrease in glucocorticoid sensitivity and, consequently, a higher inflammatory burden. As a result, the increased intensity of the inflammatory reaction may also contribute to glucocorticoid resistance as cytokines have been reported to influence glucocorticoid sensitivity in various tissues. However, this proposed mechanism does not rule out the possibility that other genetic variants in linkage disequilibrium with *BclI *polymorphism are of functional importance. As suggested by several studies, the effects found in our study for the *BclI *polymorphism could also represent a combination of endogenous effects, hypothalamic-pituitary-adrenal (HPA) axis, and exogenous effects, glucocorticoid treatment. van Rossum and coworkers demonstrated that *BclI *G-allele carriers had lower cortisol levels after dexamethasone treatment, suggesting that they are more sensitive to the feedback action of glucocorticoids on the HPA axis [22]. In addition, Rosmond and coworkers in a study of 284 Swedish men, have shown that stimulated cortisol secretion after a standardized lunch differed between the *Bcl*I genotypes, which suggests an association between the *BclI *polymorphism and regulation of the HPA axis [23]. A glucocorticoid receptor polymorphism like *BclI *described to be associated with less immune suppression might be interesting for future studies [41].

In the present study, we did not find association of the other studied *GR *polymorphisms with lung disease progression evaluated from the slopes of decline in lung function parameters. Neither did we observe association of any of the studied polymorphisms with nutritional status and glucose metabolism. Several reasons can be put forward to explain these results. They include the types and the sensitivity of the phenotypic parameters analyzed, the role of the studied polymorphisms, and the potential contribution of the studied variants with rather low allele frequencies, which may exert only moderate effects. In addition, our studied population included only young patients. Several recent reports on the possible influence of modifier genes in CF provided evidence that there are disease stage-specific effects and that these effects are certainly more difficult to detect in young patients [42].

The present report is the first investigating the impact of the *GR *polymorphisms on expression of lung disease in CF. So far, studies performed on chronic respiratory disorders have mainly focused on asthma [16]. Steroid-resistant asthmatics have a disease that fails to respond to high-dose steroid therapy, despite the fact that the obstruction of their airways is reversible in response to inhaled beta-2 agonists. Several investigators have provided data suggesting increased expression of GRβ, a splice variant of the GRα isoform that does not bind glucocorticoid ligands and is unable to transactivate glucocorticoid responsive genes [43]. In interstitial lung diseases, it is suggested that the different responses to glucocorticoids may also be the results of differences in the expression of GRα [44].

Although the results of our study require further confirmation, the findings may have important therapeutic implications. The association of *BclI *polymorphism and lung disease progression in CF gives support to the concept that specific subgroups of patients with CF may benefit from the use of glucocorticoids preferably by the inhaled route. If true, this should allow discriminatory prescribing which is of tremendous importance for several reasons. One is the constant increase in the use of inhaled glucocorticoids in patients with CF. However, as indicated above, there is little evidence to justify their routine and widespread use [14]. Although there is currently compelling results to consider anti-inflammatory molecules as a major therapeutic strategy for slowing the decline in lung function and improving survival, inhaled glucocorticoids should be selectively prescribed to patients who may benefit from them [7]. However, assessment of the effect of these molecules is difficult in CF. Consequently, inhaled glucocorticoids are often maintained at high doses for long periods despite the increasing recognition that they can lead to significant adverse effects such as adrenal suppression.

In addition to patient age, a number of limitations of our study should be raised. First, the size of the population. Although, we have studied a high enough sample size for detection of significant differences, replication in additional cohorts is required to validate the association of *BclI *polymorphisms and lung disease progression in CF. In addition, the implication of the other GR polymorphisms should be tested in larger groups of patients. Another concern relates to the retrospective collection of the data from patients' medical records, leading our study shearing the general limitations of retrospective studies. Information was collected from physicians in charge of the patients as the primary source of data. All the treatments the patients received were recorded. However, data on the use of inhaled glucocorticoids could not be adequately obtained due to the large variation in molecules, drug dosages, regimens, and duration of treatment given to the patients. In addition, the adherence of each individual to the prescribed inhaled glucocorticoids could not be ascertained.

## Conclusion

In conclusion, we report for the first time the possible association between *BclI *polymorphism of the GR gene and the progression of lung disease in CF. Identification of factors implicated in the glucocorticoid response has important implications in predicting the individual therapeutic outcome. This pharmacogenetic approach should help to optimize anti-inflammatory therapy in patients with CF.

## Competing interests

None of the authors have any commercial or other associations that might pose a conflict of interest. All of the authors are aware and agree to the content of the paper and approve its submission.

## Authors' contributions

HC, drafting the manuscript, and NN have been involved in conception and design of the study and in acquisition and interpretation of data. CC has been involved in the acquisition of the data. KC, PLR and OT have been involved in the interpretation of the data. AH-C has been involved in collecting the patients DNA and the phenotypical data. BF and JF have been involved in revisiting the manuscript. P-YB has performed all the statistical analyses. AC has been involved in conception and design of the study and in critically revisiting the manuscript.

## Financial support

Institut National de la Santé et de la Recherche Médicale, Assistance Publique-Hôpitaux de Paris, Université Pierre et Marie Curie Paris, Agence Nationale de la Recherche, Association Vaincre La Mucoviscidose, Chancellerie des Universités (Legs Poix), Association Agir Informer Contre la Mucoviscidose, GIS-Institut des Maladies Rares.
